# Front Instabilities and Invasiveness of Simulated 3D Avascular Tumors

**DOI:** 10.1371/journal.pone.0010641

**Published:** 2010-05-26

**Authors:** Nikodem J. Poplawski, Abbas Shirinifard, Ubirajara Agero, J. Scott Gens, Maciej Swat, James A. Glazier

**Affiliations:** 1 Biocomplexity Institute and Department of Physics, Indiana University, Bloomington, Indiana, United States of America; 2 Departamento de Física, Universidade Federal de Minas Gerais, Belo Horizonte, Brazil; IBM Thomas J. Watson Research Center, United States of America

## Abstract

We use the Glazier-Graner-Hogeweg model to simulate three-dimensional (*3D*), single-phenotype, avascular tumors growing in an homogeneous tissue matrix (*TM*) supplying a single limiting nutrient. We study the effects of two parameters on tumor morphology: a diffusion-limitation parameter defined as the ratio of the tumor-substrate consumption rate to the substrate-transport rate, and the tumor-TM surface tension. This initial model omits necrosis and oxidative/hypoxic metabolism effects, which can further influence tumor morphology, but our simplified model still shows significant parameter dependencies. The diffusion-limitation parameter determines whether the growing solid tumor develops a smooth (noninvasive) or fingered (invasive) interface, as in our earlier two-dimensional (*2D*) simulations. The sensitivity of 3D tumor morphology to tumor-TM surface tension increases with the size of the diffusion-limitation parameter, as in 2D. The 3D results are unexpectedly close to those in 2D. Our results therefore may justify using simpler 2D simulations of tumor growth, instead of more realistic but more computationally expensive 3D simulations. While geometrical artifacts mean that 2D sections of connected 3D tumors may be disconnected, the morphologies of 3D simulated tumors nevertheless correlate with the morphologies of their 2D sections, especially for low-surface-tension tumors, allowing the use of 2D sections to partially reconstruct medically-important 3D-tumor structures.

## Introduction

In [1] we studied the effects of nutrient limitation and surface tension in a simplified two-dimensional (*2D*) model of tumor invasion using the Glazier-Graner-Hogeweg (*GGH*) (also known as the Cellular Potts Model) [2]–[4], as implemented in the CompuCell3D (*CC3D*) modeling environment [5]–[10]. In 2D, the selection of smooth-interface (noninvasive) *vs.* fingered (invasive) growth depends on the tumor's substrate-consumption rate per unit substrate-transport rate, the *diffusion-limitation parameter* 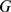, while the detailed morphology also depends on the tumor-tissue-matrix (*TM*) *surface tension* 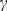. Lack of competition for nutrients promotes spherical, noninvasive tumors. Low concentrations of nutrients in the environment which cause tumor-cell competition, or cells with a very high substrate-consumption rate generate a fingering instability and irregular, invasive tumors. Our results agree with *in vitro* experiments showing that tumors branch into the surrounding tissues if the nutrient supply is too small [11], [12], and with other tumor-model predictions [13]–[18].

Our three-dimensional (*3D*) model extension of our 2D model [1], includes growing, *spatially-extended* tumor cells, surrounding TM represented as a nondiffusing field secreting nutrients, a diffusing field representing matrix-degrading enzymes (*MDE*s) that degrade TM, and a diffusing nutrient field (*substrate*) which governs the rate of tumor-cell growth. As in our 2D model we assume that all tumor cells are identical in their capacities and responses, and that the specific growth rate of tumor cells increases linearly with the local concentration of a single limiting substrate, with no concentration threshold for tumor cells to grow.

For a detailed discussion of tumor initiation and progression, see [19] and [20]. Growth of avascular *tumor spheroids* depends on diffusion of nutrients and waste products, usually limiting the spheroid's maximum size, as discussed in [1]. Tumors can be *benign* (noninvasive) or *malignant* (invasive). Most tumors are initially noninvasive and gradually become more invasive as they reduce their cell-cell adhesiveness and (often) increase their cell-extracellular-matrix (*ECM*) adhesiveness [21]. Tumor cells can secrete MDEs that degrade the ECM which maintains the integrity of normal tissues [22]–[24] and modifies the distribution in the ECM of molecules to which cells adhere, *e.g.*, fibronectin, increasing effective cell motility [22], [25]–[30]. Intrinsic cell motility can also increase during tumor progression [31]–[33].

*Hypoxia* (a shortage of oxygen) [34] activates transcription of the met proto-oncogene [12], which increases levels of the Met tyrosine kinase, a receptor for hepatocyte growth factor (*HGF*) [35]–[37]. HGF is a *scatter factor* [38] that coordinates a number of specific cytokines [39] which weaken cell-cell contacts and increase cell motility [40], [41]. Thus hypoxia indirectly enhances HGF-induced cell motility [12], [42]. Hypoxia can also induce epithelial-mesenchymal transitions (*EMT*) [43] through Notch signaling [44], one of the initial steps in metastasis, transforming nonmotile, epithelial cells into migratory and invasive cells, *e.g.*, through down-regulation of E-cadherin [45], [46].

Mathematical models of tumor growth [47] range from simple fitting of experimental data on the growth kinetics of tumor spheroids using various growth laws [48] to more complex simulations of tumor-induced angiogenesis and capillary network formation [47], [49]–[52], and tumor spreading at early [53] and later invasive stages [18], [54]–[58]. Continuum and solid-mechanics models [14]–[17], [59]–[62] consider physical forces among cells and TM, capturing tumor structure at the tissue level, but do not describe the tumor's cellular and subcellular properties, making mechanisms such as cell-cell adhesion difficult to include. Point-cell models (*cellular automata*) allow stochastic descriptions at cellular [63]–[67] and subcellular levels [68], [69] but neglect the shapes of cells. Hybrid multi-cell models combine discrete representations of individual tumor cells with continuum representations of diffusible chemicals [70]–[74] and either discrete or continuum models of the surrounding tissue. For comprehensive reviews of mathematical models of tumor growth see [75], [76] and references therein.

Three recent models of tumor growth have analyzed tumor-growth morphologies in a two-dimensional parameter phase space. The model of [15] analyzed nonlinear tumor morphological response to two nondimensional parameters representing the balance of cell death to birth and tissue mechanics (proliferation-induced pressure), the model of [16] analyzed fragmenting and compact morphologies in the nutrient-adhesion phase space, and the model of [17] analyzed the fragmenting, fingering, and compact morphologies in the nutrient-mechanics phase space.

In our recent 2D GGH simulations of growing avascular tumors [1] we found that smaller tumor-TM surface tension speeds diffusion of tumor cells and that simulated substrate-deprived tumor morphologies are more sensitive to variations in tumor-TM surface tension. These results agree with the observation that hypoxia enhances the sensitivity of tumor cell motility to scatter factors, and suggest an experimentally-testable hypothesis that HGF decreases tumor-TM surface tension, which we could measure, *e.g.*, using compression apparatus [77]–[80]. They also agree with a recent study of the analogy between branching instabilities in a growing tumor and instabilities in a drop of water impacting a solid surface, which suggested promoting tumor cell-tumor cell adhesion as a clinical strategy in oncological therapies [81].

As in [1], we do not model explicitly the haptotactic repulsion and pressure on the tumor cells from the surrounding normal tissue. Thus our simulated tumor cells move freely, which is realistic only for environments that do not constrain tumor-cell motility, such as idealized gliomas or those growing in mechanically unconfined areas, *e.g. in vitro* [61], [82]. However, the tumor-TM surface tension creates an effective hydrostatic pressure on the tumor. While not identical to a tumor growing in an elastic or viscoelastic tissue, increasing the surface tension reproduces many of the effects of increasing the rigidity of an explicitly-modeled external tissue.

Necrosis can be essential to instability mechanisms at later stages of tumor growth, destabilizing the shape of the tumor [17]. While diffusional instabilities lead to fingering morphologies, the connecting portions of the fingers experience necrosis due to prolonged hypoxia and anoxia, leading to a disconnection of the fingers and a fragmented morphology. Necrosis becomes biologically significant when hypoxia of a layer of cells surrounding the necrotic core of the tumor triggers a cascade of signals mediated by hypoxia-inducible factor-1 (*HIF-1*) [83] and vascular endothelial growth factor (*VEGF*) [84], which initiate *angiogenesis*, *i.e.* tumor vascularization, by inducing growth and extension of nearby blood vessels. Since we focus on the role of cell-cell adhesion and competition for nutrients at the tumor-TM interface where the tumor cells are alive and proliferating, as in [1], we simulate single-cell-type avascular tumors without angiogenesis, omitting necrotic and quiescent cells, which are absent at the tumor-TM interface during early stages of fingering. While necrosis certainly has a profound effect on the late-stage morphology of fingered tumors, its primary effect on the instabilities we are studying is to reduce the competition for substrate, thus changing the 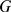 values for the onset of different instabilities. Since the degree of shift depends on details of the necrotic mechanism, we feel that studying the effects of nutrient limitation separately from the effects necrosis is clearer. We will combine the effects in a later paper. We do not model quiescence explicitly because the substrate concentration in the central regions of our simulated tumors is nearly zero, so the cells there barely grow (see Mathematical Structure of the Tumor Model), effectively behaving like quiescent cells.

In this paper we extend our 2D model of tumor-interface instabilities to more realistic 3D tumors. We find that our results for our 3D simulations agree with our 2D results, which is surprising because certain relationships, such as mutually penetrating connected structures, cannot exist in 2D. Such structures form in real tumors during neoangiogenesis [85], during which tumors recruit blood vessels from the surrounding vascular network to supply nutrients and remove waste. We need to understand the physics of instabilities in growing avascular 3D tumors before we proceed to investigate how 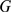 and 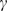 affect vascular tumors undergoing neoangiogenesis, extending the recent GGH simulations in [52].

We aim to answer two questions: 1) *what causes front instabilities and invasiveness in 3D avascular tumors?* and 2) *can we reconstruct medically-important 3D tumor structures from 2D sections?* We hypothesize that hypoxia and surface tension will have similar effects on tumor morphology in 2D and 3D simulations (which is not obvious *a priori*). We show that the diffusion-limitation parameter 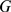 determines whether the 3D tumor has a uniform or fingered margin, while the tumor-TM surface tension 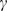 affects the detailed tumor morphology, as in 2D. We construct a 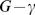 phase diagram showing the effects of these parameters on simulated 3D avascular tumors and their 2D sections. Although geometrical artifacts mean that the 2D sections of many connected 3D simulated tumors are disconnected, the morphologies of 3D simulated tumors nevertheless correlate strongly with the morphologies of their 2D sections, especially at later stages, allowing the use of 2D sections to partially reconstruct 3D tumor structures.

## Results

We can describe tumor morphologies in terms of their *sphericity* [86],
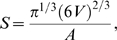
(1)where 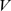 is the volume of an object and 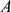 is its surface area. Sphericity is a computationally convenient measure of how round an object is for 3D images. We study the time dependence of the sphericity of simulated tumors as a function of 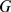 and 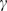. As in [1], we call structures with pronounced orientational order *dendritic*, and structures without apparent orientational order *seaweed-like* or diffusion-limited-aggregation-like (*DLA-like*). We also study how changes in 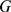 and 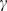 affect the time at which the middle 2D section of the simulated tumor reaches the boundary of the simulation domain, and the conditions under which the simulated tumor stops proliferating before reaching this boundary. Because 2D sections of real tumors are more convenient to analyze medically, we also measure the *circularity* [1],
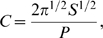
(2)where 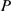 is the perimeter of an object, in our case, of the middle 2D sections of the simulated tumors, and compare it with the sphericity for the corresponding 3D images. If the sphericity of a 3D simulated tumor corresponds to the circularity of its 2D section, analysis of 2D sections of real tumors may allow us to reconstruct their 3D morphology, helping to determine whether they are invasive or not, and possibly predicting the effectiveness of antiangiogenic therapies.

**Figure 1:**
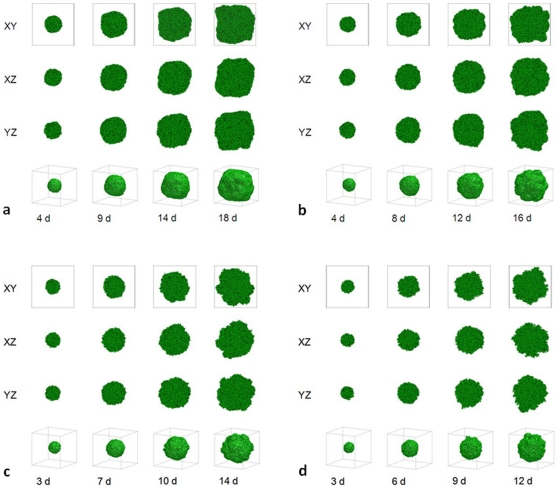
Simulated growing tumors with 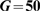. (a) 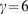. 2D sections of a 3D simulation along the XY (first row), XZ (second row) and YZ plane (third row). The developing tumor is initially spherical; it then becomes slightly irregular. Fourth row: 3D visualization of the same simulation. (b) 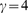. 2D sections of a 3D simulation along the XY (first row), XZ (second row) and YZ plane (third row). The developing tumor is initially spherical; it then becomes grooved. Fourth row: 3D visualization of the same simulation. (c) 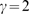. 2D sections of a 3D simulation along the XY (first row), XZ (second row) and YZ plane (third row). The developing tumor is initially spherical; it then becomes grooved. Fourth row: 3D visualization of the same simulation. (d) 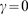. 2D sections of a 3D simulation along the XY (first row), XZ (second row) and YZ plane (third row). The developing tumor is initially spherical; it then becomes grooved with a rough surface. Fourth row: 3D visualization of the same simulation. The simulation time is indicated in days beneath each column, where 1 day = 400 MCS.

Small 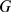 (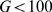) corresponds to a *growth-limited regime* [1], [87]. The substrate penetrates most of the tumor and reaches most cells, so the simulated tumor remains spherical [1]. Larger 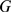 slows the growth of the tumor (since the local substrate concentration decreases in the presence of tumor cells), and diffusing substrate penetrates fewer cell diameters past the surface layer of the tumor. Initially, substrate is present throughout the tumor, which grows in all directions. As the tumor grows, its cells consume substrate and the substrate concentration develops a gradient, the concentration increasing in the radial direction away from the tumor centroid. Locally, cells near the tumor surface in protruding regions experience higher concentrations of substrate and hence grow faster than others. These cells proliferate more quickly, while the cells in the narrow valleys, where the interface between the tumor and TM lags significantly behind the furthest local radial position of the tumor, experience low concentrations of substrate and slow their growth. Figure 1 shows the time evolution of a simulated tumor with 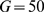 (which is near the value 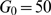 corresponding to the parameters used in [70]), for 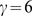 (a), 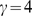 (b), 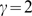 (c), and 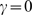 (d). The simulated tumors are dense, fast-growing and initially spherical. As they grow, their tumor-TM interfaces become slightly irregular (*grooved*) (the lower 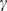, the rougher the surface of the developing tumor) but remain compact.

**Figure 2 pone-0010641-g002:**
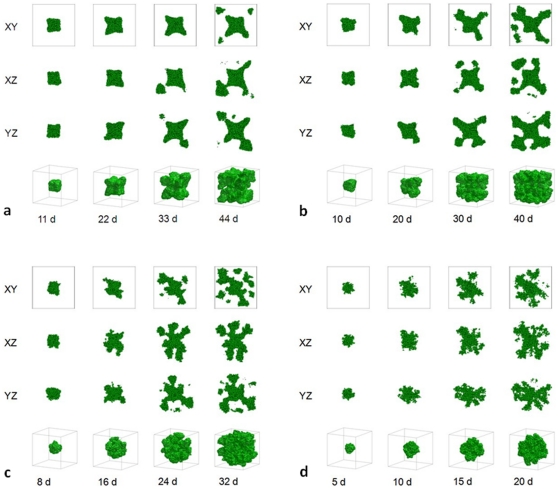
Simulated growing tumors with 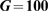. (a) 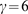. 2D sections of a 3D simulation along the XY (first row), XZ (second row) and YZ plane (third row). The developing tumor is initially compact; it then becomes dendritic. The disconnected parts in the last image connect to the backbone of the tumor out of the section plate. Fourth row: 3D visualization of the same simulation. (b) 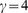. 2D sections of a 3D simulation along the XY (first row), XZ (second row) and YZ plane (third row). The developing tumor is initially compact; it then becomes dendritic. The disconnected parts in the last two images connect to the backbone of the tumor out of the section plate. Fourth row: 3D visualization of the same simulation. (c) 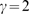. 2D sections of a 3D simulation along the XY (first row), XZ (second row) and YZ plane (third row). The developing tumor is initially compact with a rough surface; it then becomes seaweed-like. The disconnected parts in the last two images connect to the backbone of the tumor out of the section plate. Fourth row: 3D visualization of the same simulation. (d) 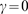. 2D sections of a 3D simulation along the XY (first row), XZ (second row) and YZ plane (third row). The developing tumor is seaweed-like with a rough surface. The disconnected parts in the images connect to the backbone of the tumor out of the section plate. Fourth row: 3D visualization of the same simulation. The simulation time is indicated in days beneath each column, where 1 day = 400 MCS.

**Figure 3 pone-0010641-g003:**
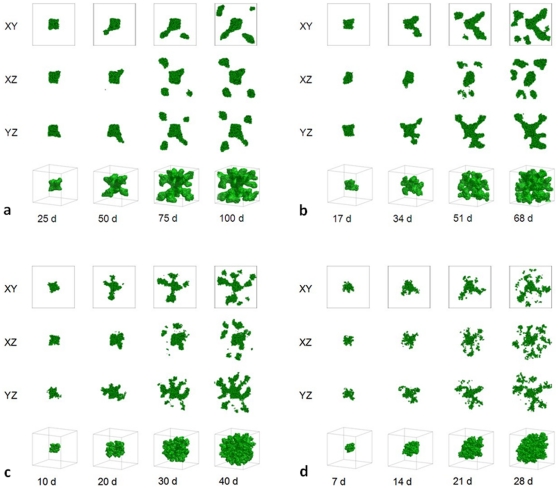
Simulated growing tumors with 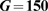. (a) 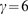. 2D sections of a 3D simulation along the XY (first row), XZ (second row) and YZ plane (third row). The developing tumor is initially compact; it then becomes dendritic. The disconnected parts in the last two images connect to the backbone of the tumor out of the section plate. Fourth row: 3D visualization of the same simulation. (b) 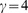. 2D sections of a 3D simulation along the XY (first row), XZ (second row) and YZ plane (third row). The developing tumor becomes dendritic. The disconnected parts in the last two images connect to the backbone of the tumor out of the section plate. Fourth row: 3D visualization of the same simulation. (c) 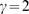. 2D sections of a 3D simulation along the XY (first row), XZ (second row) and YZ plane (third row). The developing tumor has a form intermediate between dendrite and seaweed. The disconnected parts in the images connect to the backbone of the tumor out of the section plate. Fourth row: 3D visualization of the same simulation. (d) 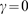. 2D sections of a 3D simulation along the XY (first row), XZ (second row) and YZ plane (third row). The developing tumor is seaweed-like. The disconnected parts in the images connect to the backbone of the tumor out of the section plate. Fourth row: 3D visualization of the same simulation. The simulation time is indicated in days beneath each column, where 1 day = 400 MCS.

Large 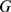 corresponds to a *transport-limited* or *diffusion-limited regime* [1], [87]. The substrate penetrates only a short distance into the tumor, so the tumor grows more slowly than for smaller values of 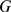. Figure 2 shows the time evolution of simulated tumors with 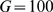, for 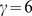 (a), 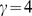 (b), 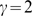 (c), and 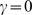 (d). For high TM-tumor surface tensions, the simulated tumors are compact and dendritic (with anisotropic branches), while for low TM-tumor surface tensions, the simulated tumors are DLA-like (with isotropic branches), as in our previous 2D simulations [1]. The effect of surface tension on morphology is more dramatic for larger 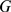, again as in 2D [1]. Figure 3 shows the time evolution of simulated tumors with 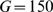, for 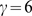 (a), 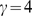 (b), 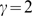 (c), and 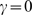 (d).

**Figure 4 pone-0010641-g004:**
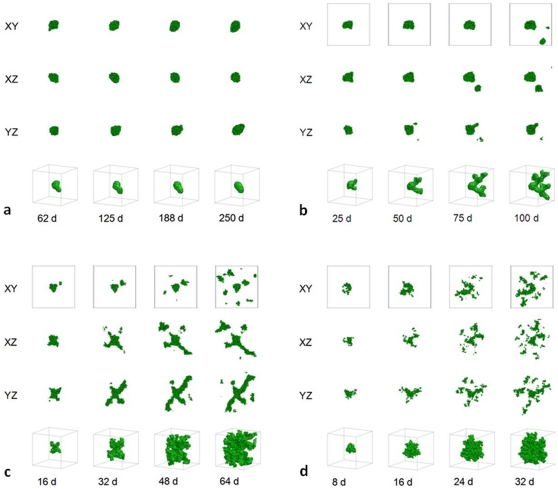
Simulated growing tumors with 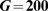. (a) 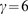. 2D sections of a 3D simulation along the XY plane. The developing tumor remains compact and ceases proliferating. We do not show 2D sections along the XZ and YZ planes because they are essentially indistinguishable from those along the XY plane. We do not show 3D visualization of the same simulation because it does not provide any new information about the simulated tumor. (b) 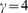. 2D sections of a 3D simulation along the XY (first row), XZ (second row) and YZ plane (third row). The developing tumor forms a truncated dendrite. The disconnected parts in the last image connect to the backbone of the tumor out of the section plate. Fourth row: 3D visualization of the same simulation. (c) 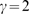. 2D sections of a 3D simulation along the XY (first row), XZ (second row) and YZ plane (third row). The developing tumor has a form intermediate between dendrite and seaweed, with thinner fingers. The disconnected parts in the images connect to the backbone of the tumor out of the section plate. Fourth row: 3D visualization of the same simulation. (d) 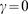. 2D sections of a 3D simulation along the XY (first row), XZ (second row) and YZ plane (third row). The developing tumor forms a seaweed. The disconnected parts in the images connect to the backbone of the tumor out of the section plate. Fourth row: 3D visualization of the same simulation. The simulation time is indicated in days beneath each column, where 1 day = 400 MCS.

At 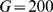 for a high surface tension, 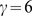, the tumor occupies a region with a high concentration of MDE that has degraded all the TM Field. TM far from the tumor still produces substrate, but the substrate is essentially exhausted at the tumor surface. The availability of nutrients is so limited that cell proliferation nearly stops; the simulated tumor does not reach the boundary of the simulation domain (Figure 4 (a)). For a lower surface tension, 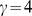, the simulated tumor does reach the boundary of the simulation domain but so many cells stop dividing that some branches of the dendritic tumor stop growing (Figure 4 (b)). Figures 4 (c) and 4 (d) show the time evolution of simulated tumors for 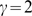 and 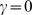, respectively. For such low surface tensions, DLA-like structures form.

**Figure 5:**
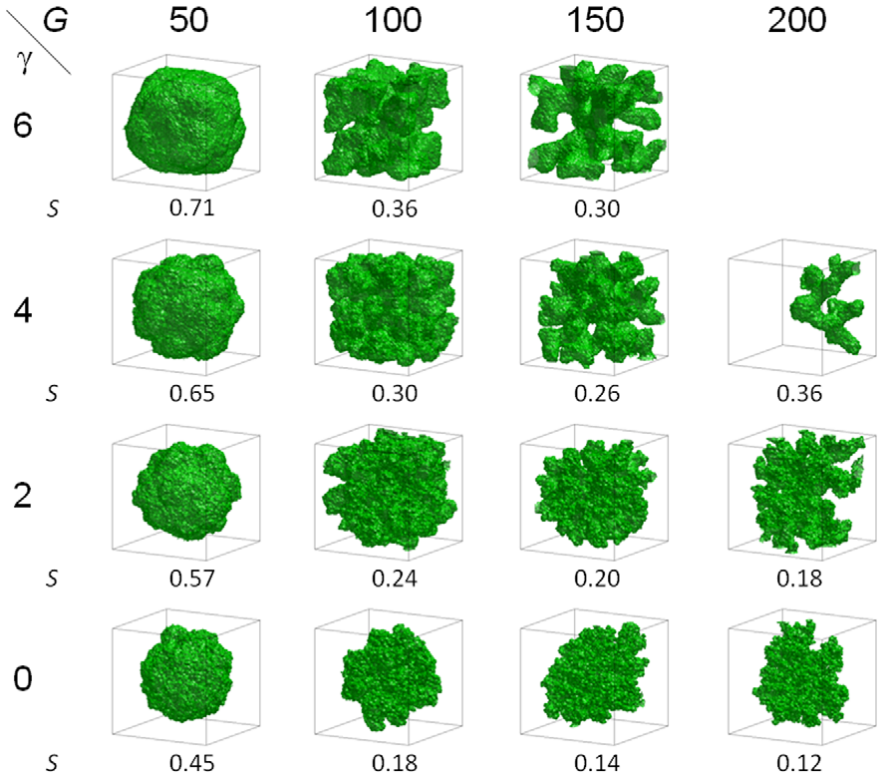
Morphologies of 3D tumors visualized in 3D and sphericity 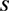 as a function of 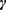 and 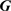, observed when the simulated tumor reaches the boundaries of the simulation domain (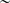
6 mm). The standard deviation for 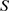 is less than 0.02. The panel for 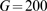 and 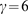 is blank because the corresponding tumor never grows to this size.

Figure 1 shows that, for small 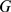, the substrate reaches most cells, which grow in all directions, no valleys form and the tumor-TM interface remains smooth. As we increase 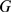, the substrate penetrates less deeply into the tumor. Cells near the tumor surface experience higher concentrations of substrate and proliferate more quickly, producing fingers, while the space between fingers fills slowly or not at all with new cells, as Figures 2–4 show. Thus the *competition* for substrate between tumor cells results in a *fingering instability* [15], [88]–[90] which generates a fingered tumor morphology [1]. Table 1 shows the mean circularity 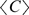, defined as the average of the circularities of 2D sections of 3D tumors taken at the midplanes XY, XZ and YZ, for different values of 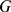 and 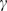 at the moment when the tumor reaches the boundaries of the simulation domain (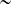
6 mm). Figure 5 summarizes the morphologies and shows the sphericity 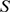 of 3D tumors for different values of 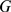 and 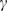 at the moment when the tumor reaches the boundaries of the simulation domain (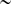
6 mm). Although the 2D sections of many simulated tumors are disconnected, the underlying 3D tumors are connected (except for 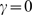 where a few cells migrate out of the backbone of the tumor). Thus the disconnectedness of tumors simulated in 2D results from the underlying physics of growth and diffusion and provides fundamental information about the growth dynamics, while the disconnectedness of 2D sections of connected 3D simulated tumors is a geometrical artifact and does not provide much information beyond indicating a rough tumor surface.

**Table 1:** The dependence on 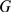 and 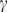 of the mean circularity 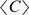 of 2D sections of the simulated 3D tumors, observed when the tumor reaches the boundaries of the simulation domain (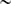
6 mm).

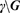	50	100	150	200
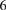	0.74 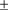 0.02	0.37 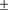 0.03	0.35 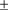 0.03	
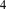	0.69 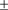 0.02	0.31 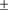 0.02	0.29 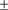 0.07	0.51 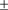 0.04
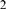	0.62 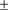 0.03	0.25 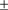 0.04	0.22 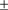 0.01	0.21 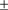 0.02
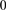	0.54 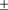 0.02	0.23 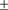 0.03	0.16 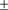 0.01	0.15 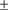 0.02

The space for 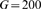 and 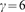 is blank because the corresponding tumor never grows to this size.

**Figure 6:**
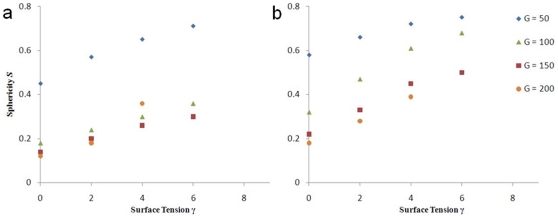
Sphericity 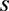 of simulated tumors as a function of 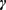 for different 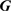. (a) When they reach the boundary of the simulation domain, (b) with 1000 Generalized Cells.

Sphericity increases with surface tension 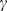 and decreases with increasing 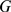 (the structure for 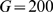 and 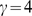 deviates from this general behavior because its growth is truncated). These dependencies of 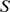 are consistent with our observation that competition for substrate favors branching instabilities while surface tension stabilizes the tumor-TM interface, as in 2D [1]. Table 2 shows how the sphericity of simulated tumors with 1000 Generalized Cells (which represent 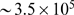 real tumor cells) depends on 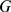 and 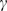. Figure 6 shows how the sphericity 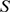 of simulated tumors, when they reach the boundaries of the simulation domain (a) or contain 1000 Generalized Cells (b), depends on surface tension 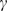 for different 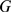. This dependence, more monotonic for a fixed number of cells than for a fixed size, suggests that the mass of the tumor, which is proportional to the number of tumor cells, is a more accurate description of the tumor stage than its size.

**Table 2:** The dependence on 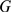 and 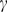 of the sphericity 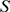 of the simulated tumors with 1000 Generalized Cells.

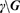	50	100	150	200
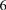	0.75	0.68	0.50	
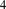	0.72	0.61	0.45	0.39
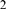	0.66	0.47	0.33	0.28
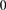	0.58	0.32	0.22	0.18

The space for 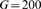 and 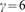 is blank because the corresponding tumor ceases to grow before reaching 1000 Generalized Cells.

Table 1 and Figure 5 show that the sphericity 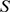 of 3D simulated tumors and the mean circularity 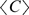 of their 2D sections differ by 0.05 or less, except for 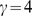 and 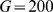 (truncated growth) and for 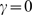 and 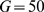. The sphericity 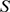 is smaller than the mean circularity 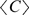, except for 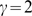 and 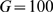. Our results indicate a close relationship between the numerical values of 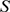 and 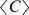 for a given tumor and provide a simple method for partial reconstruction of 3D tumor structure from 2D sections: *the sphericity of a real 3D tumor of a size on the order of a millimeter approximately equals the mean circularity of the 2D sections taken at the midplanes XY, XZ and YZ through the 3D tumor*. This partial reconstruction is the first step in determining the 3D tumor's morphology, and thus potentially has medical value in predicting its behavior.

**Figure 7:**
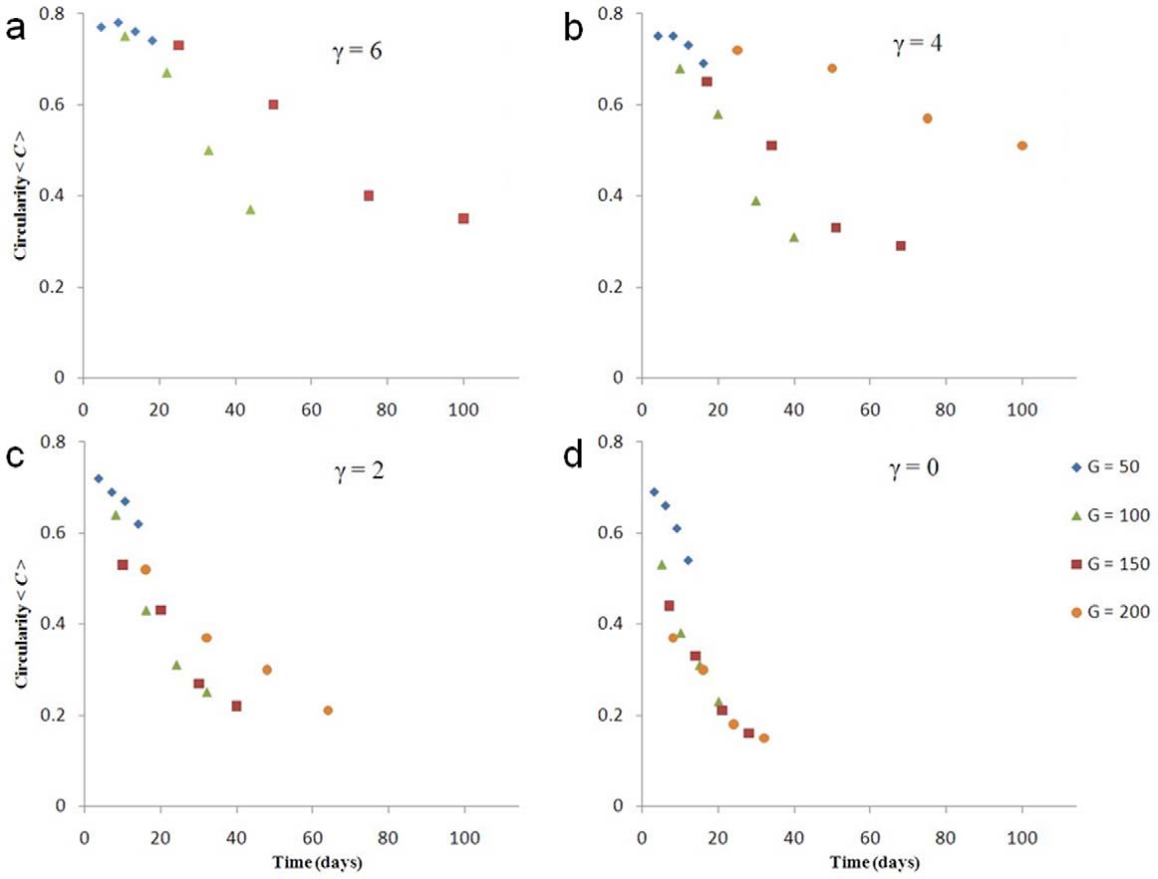
Mean circularity 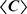 as a function of time for 2D sections of 3D simulations of tumor growth. (a) 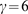, (b) 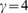, (c) 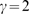, and (d) 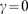.

**Figure 8:**
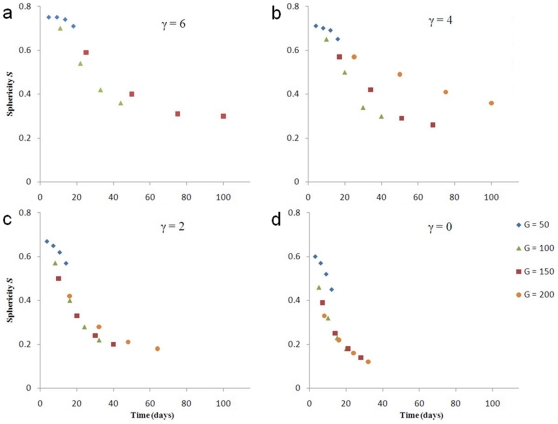
Sphericity 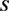 as a function of time for 3D simulations of tumor growth. (a) 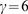, (b) 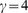, (c) 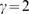, and (d) 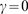.

**Figure 9:**
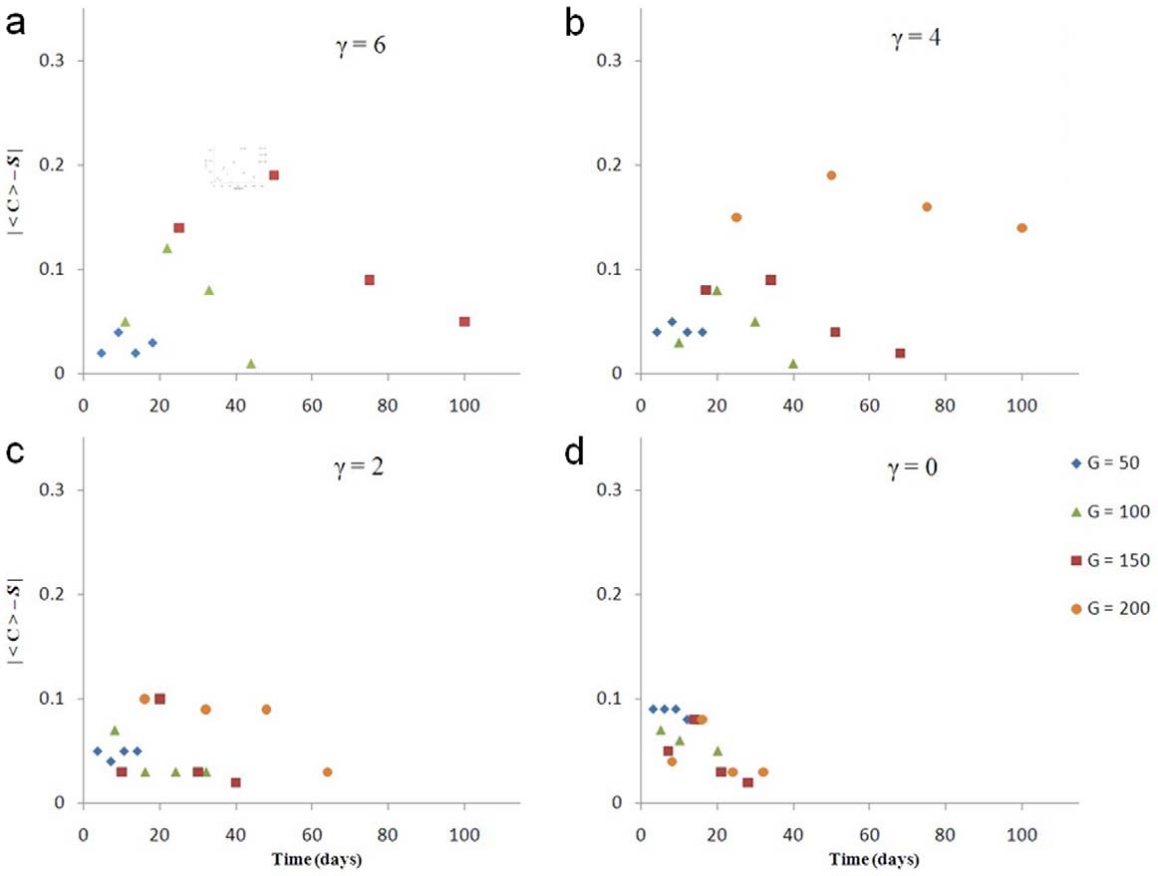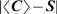 as a function of time for 3D simulations of tumor growth. (a) 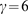, (b) 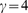, (c) 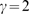, and (d) 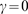.

Figures 7 and 8 show that the sphericity 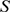 of 3D simulated tumors and the mean circularity 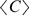 of their 2D sections both decrease in time 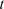 for all 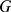 and 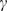. 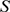 varies less with 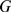 than 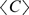 does. The 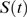 curves for 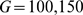 at 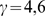, and for 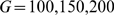 at 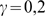 lie close to each other, indicating that 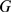 does not strongly affect how the sphericity of simulated low-surface-tension tumors changes in time. The 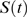 curve for 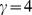 and 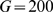 deviates from the curves for the other 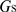
s because tumor growth is truncated. We do not show the 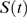 curve for 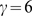 and 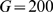 because the simulated tumor does not grow. The 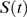 curves are more concave (have larger slopes) for lower surface tensions. The absolute value of the difference between 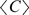 and 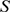 for almost all simulated tumors initially increases in time and then decreases, as Figure 9 shows (except for 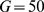, where it is not monotonic and remains small). Also, 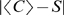 decreases with 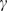. Therefore, reconstructing 3D tumor structure from 2D sections is more accurate for avascular tumors either at very early or at later stages, and is more accurate for low-surface-tension tumors which grow faster and thus are medically relevant.

Table 3 shows how the time at which the simulated tumor reaches the boundary of the simulation domain, which has a size of 6 mm, depends on 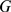 and 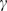. The smaller the tumor-TM surface tension, 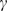, the faster the growth of the tumor, because for large 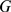, tumor cells must diffuse to find substrate to maintain their growth rather than having substrate diffuse to reach them. The smaller 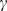, the larger the diffusion coefficient of the tumor cells [91]. Small surface tension enhances spreading of the tumor cells, so the tumor grows continuously. This result agrees with experiments showing that less adhesive tumors grow faster [43]. Table 4 shows how the time at which the simulated tumor grows to 1000 cells depends on 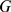 and 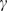.

**Table 3:** The dependence on 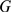 and 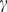 of the time (in days) at which the simulated tumor reaches the boundary of the simulation domain.

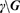	50	100	150	200
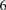	18	44	100	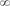
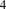	16	40	68	100
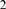	14	32	40	64
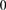	12	20	28	32

**Table 4:** The dependence on 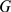 and 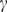 of the time (in days) at which the simulated tumor grows to 1000 Generalized Cells.

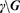	50	100	150	200
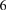	2	5	14	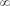
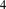	2	5	11	35
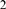	2	5	8	13
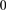	2	4	6	9

Almost all the 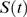 curves in Figure 8 have a quasi-Gaussian profile. Thus we can fit them to a function of form 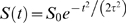, where 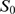 and 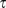 are constants. The *characteristic time* 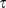 then characterizes how fast the tumor develops fingers and thus is a useful way to characterize tumor morphology dynamics. Table 5 shows 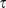 as a function of 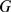 and 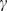. Sensitivity of simulated-tumor morphology dynamics to changes in 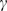 decreases with increasing 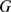 in the growth-limited regime, then increases with increasing 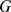 in the diffusion-limited regime, in agreement with experiments showing that hypoxia enhances the sensitivity of diffusion-limited tumors to scatter factors which increase cells' motility [12], [42].

**Table 5:** The dependence of 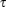 (in days) on 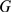 and 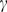.

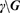	50	100	150	200
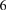	52	35	79	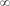
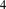	37	29	47	101
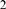	23	21	27	46
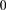	15	14	18	21

## Discussion

We have shown that whether a simulated growing 3D tumor develops a smooth or fingered interface depends primarily on 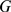, in agreement with *in vitro* experimental observations in [12], where tumor spheroids embedded in a 3D collagen matrix in hypoxic conditions developed a branched tubular structure, while in normoxic conditions they remained unbranched, confirming the previous results of [11] and recent studies employing different modeling approaches [17], [18], [92]. The transition from a smooth to fingered interface for GGH-simulated 3D avascular tumors occurs between 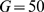 and 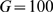, while for GGH-simulated 2D avascular tumors it occurs between 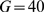 and 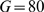 [1]. The transition regions overlap, which shows that the fingering instability is essentially dimension-independent and justifies using simpler 2D models of tumor growth instead of computationally expensive 3D models. Our GGH simulations of biofilm growth showed that changing the vertical dimension of the simulation domain, 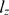, greatly affected biofilm morphology, because 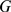 was proportional to 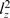 in those simulations. However, changing 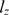 and keeping 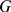 constant by changing, for instance, the background concentration of substrate did not significantly affect biofilm morphology [87]. Since the interaction between the growing tumor and the substrate does not depend on the boundaries of the simulation domain, 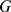 is a convenient parameter to define tumor-morphology regimes. In our simulations, we set the size of the cubic simulation domain 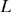 to the order of the typical size of avascular tumors, so 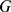 is an accurate, relative measure of how much the tumor cells compete for substrate.

Figures 1–5 show that while the tumor-TM surface tension does not affect the overall morphology significantly for low 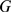, its effect grows for higher 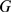. For low 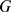, the tumor cells near the tumor-TM interface grow fast enough to find more substrate. For larger 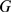, they grow more slowly, and in order to maintain their growth, they must migrate to reach substrate. The results of our 3D simulations agree with hypoxia's observed enhancement of the sensitivity of tumor cell motility to scatter factors, and support the hypothesis we suggested in [1] that HGF decreases tumor-TM surface tension.

2D simulated tumors [1] were partially disconnected for 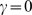 and for larger 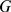. Our 3D simulated tumors always remain connected (except again for 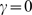 where a few cells migrate out of the backbone of the tumor), although their 2D sections appear disconnected in most cases. The ratio 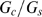, where 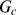 is the 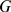 at which simulated tumors at high surface tensions cease to grow or produce truncated dendrites and 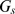 is the 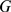 at which simulated tumors are roughly spherical, is about 5–7 in both 2D [1] and 3D. Thus the range in 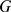 which spans all morphological regimes is very similar in 3D and 2D, *i.e.* 3D simulated tumors are as sensitive to competition for substrate as 2D tumors, which again justifies using simpler 2D models of tumor growth instead of more realistic, but computationally expensive 3D models.

While the diffusion-limitation parameter 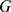 determines whether the tumor has a uniform or fingered margin, the tumor-TM surface tension 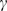 affects the detailed tumor morphology. These effects are also visible in 2D sections of simulated 3D tumors. Also as in 2D, the sensitivity of 3D tumor morphology to tumor-TM surface tension increases with 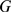, causing a directional-solidification-like transition at high 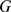 between dendritic structures, produced when the tumor-TM surface tension 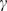 is high, and seaweed-like or DLA-like structures, produced when 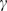 is low. Thus our 3D results support the idea, suggested in [12] and supported by our 2D simulations [1] and several other studies [16], [93], [94], that we need to therapeutically suppress cell motility and increase tumor cell-tumor cell adhesion when targeting tumor angiogenesis, in order to prevent the spread of tumor cells because of substrate deprivation.

Using a cubic lattice for our GGH simulations may introduce artifacts related to anisotropic spatial evolution of the simulated tumor, which can influence high-rank tensor observables. Such artifacts are of limited significance in our simulations, but do appear to a limited extent in Figure 1 (a), and more prevalently in Figures 2 (a), 2 (b), 3 (a) and 3 (b). In general, anisotropy is significant for large values of tumor-TM surface tension (large 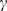), though the anisotropy does not appear to affect our general results. In these cases, we plan to cross-check our conclusions concerning orientational observables, such as dendritic orientation, with simulations on a higher-symmetry hexagonal lattice.

As in [1], we coarse-grained tumor cells to speed our simulations. Thus our Generalized Cells represent tumor-cell clusters, describing the averaged behavior of many cells. Although our model discretizes tumors at approximately the same spatial resolution as continuum methods, it introduces surface energy and cell adhesion in a correct, physical manner. Moreover, the inclusion of cells as extended and deformable objects will allow us to study in the future the effects of elasticity on simulated tumor morphology.

**Figure 10:**
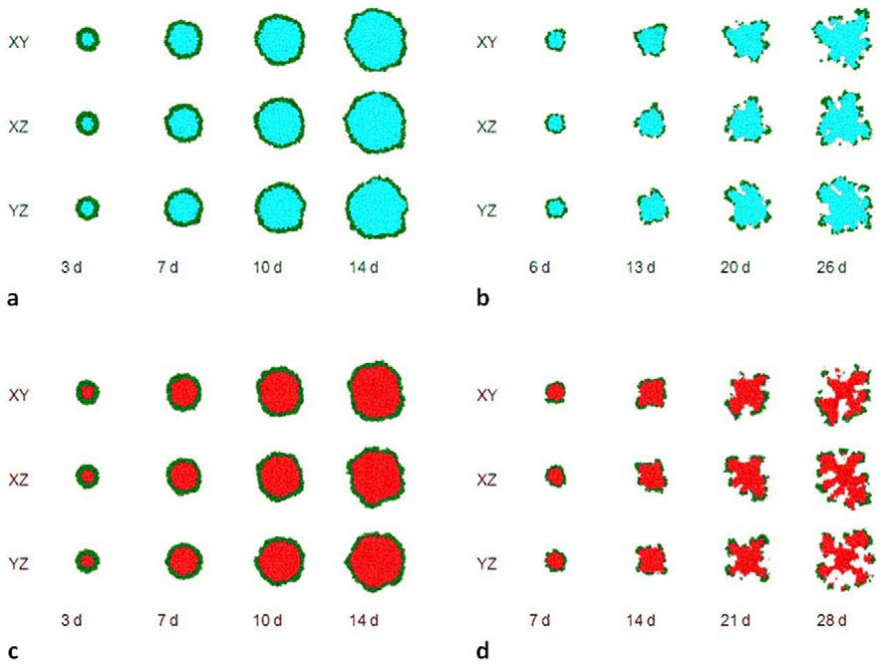
Simulated growing tumors with quiescence or necrosis for 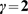. (a) 2D sections of 3D simulations with quiescence with 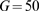. (b) 2D sections of 3D simulations with quiescence with 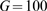. Green - proliferating cells, blue - quiescent cells. (c) 2D sections of 3D simulations with necrosis with 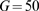. (d) 2D sections of 3D simulations with necrosis with 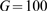. Green - proliferating cells, red - necrotic cells.

In our study of the effects of 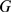 and 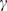 on tumor growth we did not include quiescence and necrosis explicitly. Because the substrate concentration in the central regions of our simulated tumors is nearly zero, we would expect the cells there to behave like quiescent cells (no growth). We repeated our simulations with quiescence for 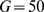 and 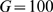, both with 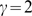. To simulate quiescence, we impose a rule that a tumor cell stops growing, consuming substrate and producing MDE if the substrate concentration inside the cell drops below a threshold 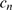. Figures 10 (a) and 10 (b) show the morphologies of 2D sections of 3D tumors with quiescence for 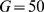 and 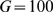, respectively. These morphologies are slightly more compact than the corresponding structures in Figures 1 (c) and Figure 2 (c). The tumors reach the boundaries of the simulation domain (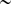
6 mm) after 14 days (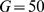) and 26 days (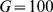). Their mean circularities at the moment when the tumors reach the boundaries of the simulation domain are slightly larger than without quiescence, 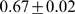 and 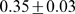. This difference arises because quiescent cells do not grow and do not consume substrate, reducing the competition for substrate. The net effect is slightly faster growth (for 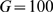) than without quiescence and morphologies corresponding to smaller values of 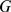.

We also repeated our simulations with necrosis for 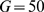 and 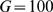, both with 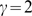. To simulate necrosis, we impose a rule that a tumor cell stops consuming substrate and producing MDE and shrinks (grows with a negative growth rate 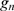) if the substrate concentration inside the cell drops below 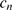. Figures 10 (c) and 10 (d) show the morphologies of 2D sections of 3D tumors with necrosis for 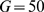 and 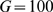, respectively. These morphologies do not differ appreciably from the corresponding structures in Figure 1 (c) and Figure 2 (c). The tumors reach the boundaries of the simulation domain (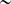
6 mm) after 14 days (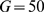) and 28 days (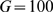). Their mean circularities at the moment when the tumors reach the boundaries of the simulation domain are slightly larger than without necrosis, 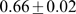 and 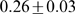. This difference arises because necrotic cells do not grow and do not consume substrate, reducing the competition for substrate. The net effect is slightly faster growth (for 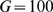) than without necrosis (but slower than with quiescence) and morphologies corresponding to slightly smaller values of 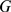. In addition, as we would expect, at late times, the tumors are more fragmented than without necrosis, which could affect their biomedically significant degree of invasiveness. However, at early times, tumor morphologies are essentially indistinguishable with and without necrosis. We will examine effects of quiescence and necrosis in more detail in a future paper.

While we recognize that most tumors are much more complex than our simple simulations, our goal was to understand the physics of the initial phase of the fingering instability as a function of the tumor-cell adhesivity and substrate consumption rate. We have shown that in 3D simulations, which are more expensive computationally, the physics of front instabilities and invasiveness in GGH-simulated tumors is the same as in 2D simulations (which was not obvious *a priori*). Therefore, our results justify using simpler 2D models of tumor growth instead of 3D models in some cases. We also found that the sphericity of 3D simulated tumors of a size on the order of a few mm (the typical size that avascular tumors reach) correlates strongly with the mean circularity of their 2D sections, especially for faster-growing, low-surface-tension tumors. Our results suggest that analyzing 2D sections of real avascular tumors at later stages should allow us to reconstruct the morphology of the underlying 3D tumors for use in tumor staging and guidance of therapeutic choices.

In future work, we will check for lattice artifacts and coarse-graining effects, and study the effects of necrosis and hypoxia-dependent growth rates on the morphological instability of growing tumors. We also plan to study the sensitivity of the tumor morphology to the other parameters in the simulations and the variability of morphology from replica to replica.

## Methods

### Mathematical Structure of the Tumor Model

We have discussed our model in detail in [1] and review it very briefly here. In our simplified solid, avascular tumor model, cells are spatially extended and deformable, move, adhere to each other, consume substrate, grow at a rate proportional to the local substrate concentration, divide when their volume doubles, and secrete MDE. Our choice of biological mechanisms follows our recent 2D model [1], which simplifies the HDC model of [70].

We use the GGH model [2]–[4] to represent tumor cells. As in [1], [70], we include three fields: a TM field representing a homogenized version of the cells and ECM of the normal tissue surrounding the tumor, an MDE field produced by tumor cells, which degrades the TM field, and a substrate field representing the concentration of a substrate limiting tumor-cell growth. The equations for these fields are the same as in [1]. MDE (denoted by 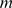, which ranges between 0 and 1) degrades TM (denoted by 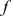, which ranges between 0 and 1) according to [95], [96] (equation (1) in [1]):
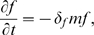
(3)where 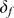 is a positive constant. Degradation does not consume MDE and MDE does not decay. However, the maximum MDE concentration is 1, which effectively imposes a decay in regions of high concentration. Tumor cells produce MDE at a constant rate 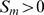 (the rate of MDE production per tumor cell); MDE then diffuses uniformly (equation (2) in [1]):
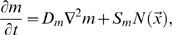
(4)where 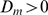 is the diffusion constant of MDE. 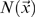 equals 1 inside tumor cells, otherwise it is 0. To model the transport of nutrient in surrounding normal tissue, TM produces substrate at a constant rate per unit density. We denote the substrate concentration 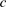 as a fraction of the maximum soluble concentration 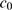, so we require 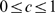. The substrate diffuses and is consumed by the tumor cells at a constant rate per cell (equation (3) in [1]). Including saturation:
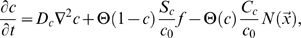
(5)where 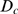, 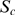 and 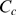 are positive constants representing respectively the substrate-diffusion constant, the substrate-production rate per unit TM and the substrate-consumption rate per tumor cell, and 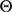 is the Heaviside step function. The substrate-consumption rate for the normalized substrate field is:
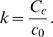
(6)Initially 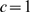, 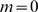 and 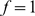 everywhere.

In [1] we showed that 2D-tumor morphology depended mainly on a single parameter, the nondimensional ratio of the maximum tumor-growth rate to the maximum substrate-transport rate: the *Diffusion-Limitation Parameter* (equation (8) in [1]):
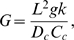
(7)where 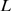 is the size of the simulation domain and 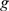 is the *maximum specific growth rate* (amount of new tumor produced per unit time per unit tumor). We vary 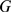 by varying 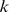. Cells grow by increasing their volume 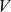 at a rate proportional to the local substrate concentration.

### GGH Implementation of the Tumor Model

For a detailed description of GGH simulations, see [1] and [4]. For additional information on CC3D and open-source downloads of CC3D software for Windows, Mac OSX and Linux platforms, please visit: http://www.compucell3d.org/.

In our simulation, *Generalized Cells* are spatially-extended domains, which represent either tumor cells or non-tumor tissue and reside on a single 3D, square *Cell Lattice* [4], [97]. Generalized Cells carry state descriptors, *e.g.*, cells' target volumes and volumes at which mitosis occurs. *Fields* are continuously-variable concentrations, each of which resides on its own lattice, here diffusing MDE and substrate, and nondiffusing TM, evolving according to equations (3), (4) and (5). The *Effective Energy* creates forces which determine a Generalized Cell's shape, motility, adhesion and response to extracellular signals [4].

We denote the unique index of a Generalized Cell by 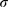, where the value at a Cell-Lattice site (*voxel*) 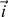 is 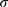 if this site lies in Generalized Cell 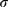. Each Generalized Cell has an associated *Generalized-Cell type* 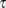. In our model, *TM* denotes tissue medium and *t* tumor cells.

The Effective Energy 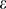 in our tumor simulations includes three terms [1]–[4]:
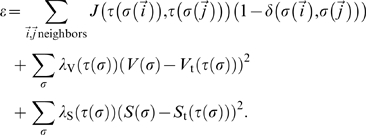
(8)The first term describes the surface adhesion between Generalized Cells in terms of *Contact Energies* 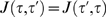 [1]–[4]. The units of 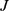 in 3D are energy/unit boundary surface area. We use a fourth-neighbor interaction range (32 neighbors for each voxel) to calculate the Contact Energies, which reduces Cell-Lattice anisotropy effects [98]. The second term constrains the Generalized Cells' volumes; 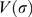 is the volume in Cell-Lattice sites of Generalized Cell 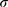, 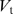 its *Target Volume*, and 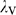 its inverse compressibility. The third term represents the elasticity of a cell membrane; 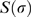 is the surface area of Generalized Cell 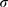, 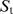 its *Target Surface Area*, and 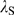 its inverse membrane elasticity. We model TM as one unconstrained Generalized Cell: 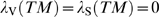.

We define the tumor *surface tension* 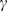 in terms of the Contact Energies 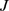 [2],[3]:
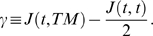
(9)The surface tension controls the tendency of tumor cells to disperse or cluster.

To model cell motility, the Cell Lattice evolves through stochastic attempts by Generalized Cells to extend their boundaries into neighboring Cell-Lattice sites, slightly displacing the Generalized Cells which currently occupy those sites [2]–[4]. At each step, we randomly select a Cell-Lattice site 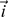 and attempt to change its index from 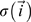 to the index 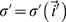 of a Cell-Lattice site 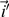 randomly chosen in its fourth-order neighborhood. If the difference in Effective Energy produced by the change 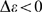 then we accept the change. If 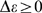 then we accept the change with a probability 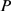:
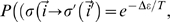
(10)where 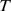 represents the cell's intrinsic cytoskeletally-driven *mot ility*. One *Monte Carlo Step* (*MCS*) corresponds to 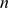 attempts, where 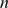 is the total number of Cell-Lattice sites.

The dimension of our Cell Lattice in voxels is 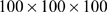. In our simulations, each simulated tumor cell (Generalized Cell of type 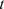) initially occupies a 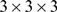 voxel cube. The initial 3D configuration of these simulations consists of 8 Generalized Cells of type 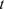 forming a cube in the middle of the simulation domain. We set the linear size of 1 voxel to 60 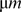, which is on the order of the scale in [1], so the size of the simulation domain corresponds to 6 mm. Therefore the linear size of 1 simulated tumor cell is 180 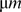, which is about 7 times greater than the size of real tumor cells [70], [99], [100], so 1 simulated tumor cell represents 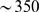 real tumor cells. Since the size of tumor cells is much smaller than the substrate-penetration and capillary lengths (see [1]), the cell size is not critical. Coarse-graining the cells greatly speeds our simulations. Since 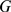 is dimensionless, its value is independent of our choice of length scale.

We simulate growth of tumor cells by increasing their 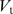 at a rate proportional to the local limiting-substrate concentration 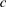 at the cell's center of mass [1], [52], [87], [101]:
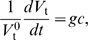
(11)where 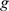 is a growth rate. For tumor cells, the initial 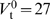. When a tumor cell reaches the *doubling volume* 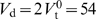, it divides and splits along a random axis into two tumor cells (of the same phenotype) with Target Volumes 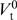 [97]. To prevent growing cells from changing shape, we adjust 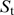 so that the nondimensional ratio 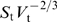 remains constant, *i.e.* 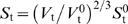. We set 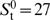, which produces roughly spherical cells. Since we do not set a substrate-concentration threshold for cells to grow, all cells can proliferate. However, the substrate concentration in the inner part of the growing tumor spheroid is nearly zero. Thus only tumor cells near the surface of the spheroid grow appreciably, while cells in the middle of the spheroid effectively do not grow during the duration of the simulation.

As in [1], we define three Fields: 1) diffusible substrate 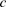, 2) diffusing MDE 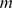, 3) nondiffusing TM 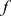. These Fields can have nonzero values at each point simultaneously and co-occupy space with cells.

Tumor cells produce MDE at a constant rate at the cell's center of mass. Tumor cells absorb substrate at their respective centers of mass. Substrate and MDE diffuse uniformly on their Field lattices using a forward-Euler algorithm. If 1 voxel corresponds to 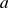 meters and 1 MCS to 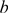 seconds then, for example, 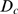 (in 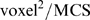) relates to the physical diffusion constant of substrate 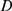 (in 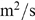) via: 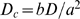. In our simulations, we use *no-flux* boundary conditions at all Field edges.

### Parameter Values

To the best of our knowledge, no one has measured the adhesion parameters for a tumor cell line, although measurement should be possible [77]–[80]. Because the Contact Energy between two Cell-Lattice sites that belong to the same Generalized Cell is defined to be zero, we set all 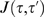 positive to prevent Generalized Cells from dissociating. We also require 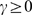 to keep the tumor cells from floating off into the TM spontaneously. Following our previous paper, our simulations use 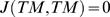 and 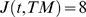, and vary 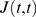 from 4, for which 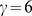, to 16, for which 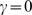. For 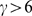, the simulated morphologies do not differ much from those for 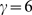. We set 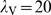 and 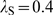, which prevents Generalized Cells from nonbiological disappearing or freezing.

As in our earlier paper, we take 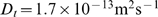 and 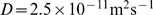. For 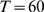, the diffusion constant for our simulated tumor cells is about 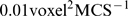, as in our previous 2D simulations, so 400 MCS corresponds to approximately 1 day and 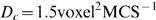.

The remaining parameters come from [1]: 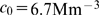, 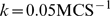 (which we denote 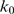), 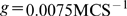, 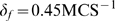, 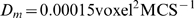, 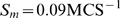, and 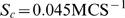. We define the production and consumption parameters per Generalized Cell, so they are the same for both 2D and 3D. We also set 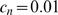 and 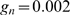. Equation (7) gives, for 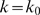, 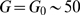. Since MDE diffusion is very slow and TM degradation by MDE is strong, 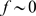 at all voxels occupied by tumor cells and 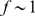 at all voxels occupied by TM.

We vary 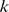 from 0.05 to 0.2, corresponding approximately to varying 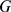 from 50 to 200, which covers the complete range of possible simulated-tumor morphologies. Smaller values of 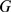 produce the same patterns as 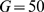 and values of 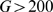 greatly slow or even halt tumor-cell proliferation. Table 6 lists our model parameters.

**Table 6:** Parameter values in our 3D simulations of tumor growth.

1 MCS	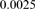 day
1 voxel	60 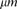
Diffusion-limitation parameter 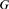	50–200
Tumor-TM surface tension 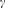	0–6
Tumor-TM Contact Energy	8
Tumor-cell motility 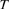	60
Tumor-cell doubling volume 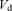	54
Tumor inverse compressibility 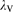	20
Tumor inverse membrane elasticity 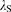	0.4
Tumor-cell shape parameter 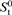	27
Substrate diffusion constant 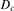	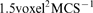
TM degradation rate 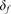	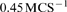
MDE diffusion constant 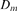	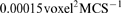
MDE production rate 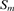	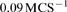
Substrate production rate 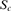	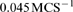
Tumor-cell growth rate 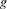	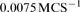
